# Sexual Reproduction *via* a 1-Aminocyclopropane-1-Carboxylic Acid-Dependent Pathway Through Redox Modulation in the Marine Red Alga *Pyropia yezoensis* (Rhodophyta)

**DOI:** 10.3389/fpls.2020.00060

**Published:** 2020-02-12

**Authors:** Toshiki Uji, Harune Endo, Hiroyuki Mizuta

**Affiliations:** Laboratory of Aquaculture Genetics and Genomics, Division of Marine Life Science, Faculty of Fisheries Sciences, Hokkaido University, Hakodate, Japan

**Keywords:** *Pyropia*, red algae, sexual reproduction, 1-aminocylopropane-1-carboxylic acid, redox signalling, ethylene, plant hormone

## Abstract

The transition from the vegetative to sexually reproductive phase is the most dynamic change to occur during a plant's life cycle. In the present study, we showed that the ethylene precursor 1-aminocylopropane-1-carboxylic acid (ACC) induces sexual reproduction in the marine red alga *Pyropia yezoensis* independently from ethylene. Exogenous application of ACC, which contains a three membered carbocyclic ring, promoted the formation of spermatia and carporspores in gametophytes, whereas ethephon, an ethylene-releasing compound, did not stimulate sexual reproduction. In addition, an ACC analog, 1-aminocyclobutane-1-carboxylic acid (ACBC), which contains a four membered carbocyclic ring, promoted sexual reproduction and enhanced tolerance to oxidative stress in the same manner as ACC, but 1-aminocyclopentane-1-carboxylic acid (cycloleucine; which contains a cyclopentane ring) did not. The application of ACC increased the generation of reactive oxygen species (ROS) and induced the expression of *PyRboh* gene encoding NADPH oxidase. ACC also stimulated the synthesis of ascorbate (AsA) by inducing transcripts of *PyGalLDH*, which encodes galactono-1,4-lactone dehydrogenase, the catalyst for the final enzymatic step of the AsA biosynthetic pathway. Conversely, ACC caused a decrease in the synthesis of glutathione (GSH) by repressing transcripts of *PyGCL*, which encodes glutamate cysteine ligase, the catalyst for the rate-limiting step in the formation of GSH. These results suggest a possible role played by ACC as a signaling molecule independent from ethylene in the regulation of sexual reproduction through alterations to the redox state in *P. yezoensis*.

## Introduction

Ethylene is a gaseous plant hormone with a plethora of effects on plant growth, development, and stress responses during events such as fruit ripening, senescence, flower development, sex determination, and pathogen attack (Lin et al., 2009; Van De Poel et al., 2015). Ethylene is synthesized through the conversion of methionine to 1-aminocylopropane-1-carboxylic acid (ACC) by ACC synthase (ACS), which acts as the enzyme for the rate-limiting step in the ethylene biosynthetic pathway. In the subsequent step, ACC is catalyzed by ACC oxidase in a reaction that converts ACC to ethylene (Yang and Hoffman, 1984). ACC has been widely used to replace ethylene treatment, because exogenous application of ACC can greatly increase ethylene production in plants (Elías et al., 2018; Sun et al., 2019).

In addition to ACC's function as the immediate precursor of ethylene, recent studies have suggested that it acts as a signaling molecule in *Arabidopsis thaliana* to regulate plant development and growth independently from ethylene (Yoon and Kieber, 2013; Van De Poel and Van Der Straeten, 2014; Vanderstraeten and Van Der Straeten, 2017). For example, Xu et al. (2008) showed that the mutant of two leucine-rich repeat receptor kinases, *fei1 fei2*, which displays a severe defect in anisotropic root growth due to decreased cellulose microfiber content in the cell wall at the root tip, was not affected by ethylene signaling, but could be restored by ethylene biosynthesis inhibitors. Similarly, Tsang et al. (2011) reported that reduced root cell elongation induced by isoxaben, a cellulose biosynthesis inhibitor, was suppressed by ethylene biosynthesis inhibitors, but not by inhibiting the ethylene response pathway. Further evidence of a role for ACC as a signaling molecule came from analyzing the loss of function of the ACS gene family in *A. thaliana* (Tsuchisaka et al., 2009). The null ACS mutant displayed embryo lethality, in contrast to the viability observed in null mutations of key components in ethylene signaling (Alonso et al., 1999; Tsuchisaka et al., 2009). These phenotypic differences between ethylene biosynthesis and signaling mutants suggest that an ACC signal is required for embryo development in *Arabidopsis* independently of ethylene signaling. Further investigation revealed that ACC, but not ethylene, positively modulates the terminal division of guard mother cells in *A. thaliana* (Yin et al., 2019). These results demonstrate that an ACC-dependent pathway is responsible for development in higher plants.

Although the signal transduction pathways for ACC remain obscure, the majority of plant hormones are highly integrated with redox or reactive oxygen species (ROS)—mediated signaling, thereby allowing plants to regulate developmental process and adaptive responses to environmental cues through modulation of protein activity or gene expression (Schmidt and Schippers, 2015; Xia et al., 2015). ROS are produced by different enzymatic systems, some of which involve NADPH oxidases, also known as respiratory burst oxidase homologs (Rbohs) in plants (Marino et al., 2012). In addition, the control of ROS is accomplished through the ascorbate-glutathione (AsA-GSH) pathway, which comprises two antioxidants, AsA and GSH, and four enzymes, ascorbate peroxidase (APX), monodehydroascorbate reductase (MDHAR), dehydroascorbate reductase (DHAR), and glutathione reductase (GR) (Pandey et al., 2015). The AsA-GSH cycle not only regulates the redox balance to protect against oxidative stress, but also plays an important role in plant developmental processes (Foyer and Noctor, 2011).

The red alga *Pyropia* (formerly *Porphyra*) belongs to the order Bangiales (Bangiophyceae), which represents an ancient lineage with fossil records that provide evidence for sexual reproduction dating back 1.2 billion years (Butterfield, 2000). Thus, elucidating the regulatory mechanisms involved in the sexual reproduction of *Pyropia* appears to be important to the understanding of eukaryotic evolution. During the sexual life cycle of *Pyropia*, the blade gametophytes bear nonflagellated male (spermatia) and female (carpogonia) gametes on the gametophytes. Fertilization occurs when the female gametes are retained on the gametophytes and successive cell divisions produce clones of the zygote, called carpospores, that grow into filamentous sporophytes (Blouin et al., 2011). A recent study on the monoecious species *Pyropia yezoensis* demonstrated that the application of ACC-induced gametogenesis and enhanced both the antioxidant capacity and the production of ethylene (Uji et al., 2016). Similarly, exogenous ACC dramatically promoted spermatogenesis and parthenogenesis in males and females, respectively, in the dioecious species *P. pseudolinearis* (Yanagisawa et al., 2019).

**Figure 1:**
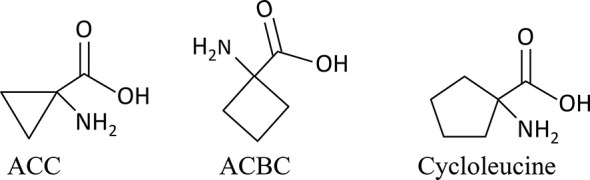
Structural formulas of 1-aminocylopropane-1-carboxylic acid (ACC) and the analogs used in this study.

In the present study, to clarify whether ACC acts as a signaling substance during sexual reproduction in red algae, we investigated the effect of ethephon, ACC, and two ACC analogs, 1-aminocyclobutane-1-carboxylic acid (ACBC) and 1-aminocyclopentane-1-carboxylic acid (cycloleucine; **Figure 1**) on growth, gametogenesis, and tolerance to oxidative stress in *P. yezoensis*. In addition, we examined the correlation between ACC and redox signaling during sexual reproduction. These findings will open up the possibility of revealing a role for ACC-ROS crosstalk, which acts independently from ethylene, in the regulation of sexual reproduction in red algae.

## Materials and Methods

### Algal Materials and Chemical Treatments

The leafy gametophytes of *P. yezoensis* strain TU-1 were cultured in a medium of sterile vitamin-free Provasoli's enriched seawater (PES; Provasoli, 1968) under conditions described previously (Uji et al., 2016). For the comparative experiment on the effects of ethephon and ACC, five individual vegetative gametophytes (ca. 20-mm blade length) were cultured in airtight glass flasks (200-ml volume) with silicone rubber stoppers and 100-ml media containing 0-, 50-, or 500-μM ACC (Tokyo Chemical Industry, Tokyo, Japan), or 500-μM ethephon (FUJIFILM Wako Pure Chemical Corporation, Osaka, Japan) without aeration at 15°C under a photoperiod regime of 10-h light:14-h dark using cool-white fluorescent lamps at 60-μmol photons m^−2^s^−1^. After treatment with ACC or ethephon for 7 days without aeration, thalli were transferred into glass flasks containing 100-ml media without ACC or ethephon under the same culture conditions but with aeration. After 7 days of culturing with aeration, the ratio of gametophytes forming clusters of spermatangia to total gametophytes was determined by counting the number of under a Leica DM 5000 B microscope, because carpogonium from *P. yezoensis* are almost indistinguishable from vegetative cells, which is in contrast to the colorless spermatangia. The numbers of discharged carpospores attached to two pieces of glass (20 mm × 25 mm) placed on the bottom of the culture flask were counted under a microscope. The growth rate was calculated as the mean percentage of length increase per day using the following formula: Growth rate = [100(BLt − BL0)/BL0]/t, BL0 = initial blade length, BLt = blade length at culture time, t = culture time.

The vegetative gametophytes were also exposed to ACC analogs, ACBC, and cycloleucine. Five individual vegetative gametophytes were cultured in glass flasks (150-ml volume) with 100-ml media containing 0-, 50-, or 500-μM ACBC (Sigma-Aldrich Co. LLC., USA.), 50- or 500-μM cycloleucine (Tokyo Chemical Industry), with aeration under the culture conditions described above. After 10 days of treatment with ACC analogs, the number of gametophytes that formed clusters of spermatangia was counted under a microscope and the growth rate was calculated. Data are expressed as means ± SD of four independent experiments with five thalli for each condition.

### Oxidative Stress Treatment

To examine the effect of ACC analogs on oxidative stress tolerance, vegetative gametophytes that had been grown to 20–40-mm blade length were cultured in glass flasks with 100-ml PES media containing 0- or 500-μM ACC, ACBC, or cycloleucine for 7 days under the culture conditions described above. For the oxidative stress treatments, thalli treated with or without ACC or ACC analogs were transferred into a Petri dish with PES medium containing 2-mM hydrogen peroxide (H_2_O_2_, Kanto Chemical Co., Inc., Japan) for 1 week. Cell mortality was measured by counting the living cells (brownish red color) and dead cells (yellowish white color) using six photographs which were taken of the upper, middle, and basal parts of each gametophyte. Data are expressed as means ± SD of three independent experiments with five thalli for each condition.

### ROS Quantification

The vegetative gametophytes (0.03-g fresh weight; FW) cultured in 100-ml medium containing 500-μM ACC were incubated at 15°C in a Petri dish with 10-ml PES medium containing 5-μM 2',7'-dichlorofluorescein diacetate (DCFH-DA, FUJIFILM Wako Pure Chemical Corporation) for 1 h. After incubation, algal thalli were rinsed in seawater, blotted dry, and ground in a mortar with a pestle under liquid nitrogen and extracted in 1 ml of 40-mM Tris-HCl buffer at pH 7.0. The homogenate was centrifuged at 10,000 ×*g* for 10 min and 500 μl of the supernatant was diluted to 2.5 ml with Tris-HCl buffer and used to measure fluorescence at 488 nm (excitation wavelength) and 525 nm (emission wavelength) with a spectrofluorometer (FB-750, Jasco, Tokyo, Japan). The data are presented as means ± SD of three independent experiments.

### Measurements of Glutathione and Ascorbate Levels

The ascorbate (AsA) and glutathione (GSH) contents were assayed according to the methods described by Ratkevicius et al. (2003). For the assay of AsA and dehydroascorbate (DHA), 0.05-g samples (FW) were ground in liquid nitrogen with a pestle and mortar. The homogenates were added to 500 μl of 5% (w/v) trichloroacetic acid and centrifuged at 4°C for 10 min at 15,000 ×*g*. AsA was detected by adding 100 µl of the supernatant to a reaction mixture containing 2% (w/v) trichloroacetic acid, 8.8% ortho‐phosphoric acid, 0.5% α, α′‐dipyridyl, and 10 mM ferric chloride in a final volume of 1 ml. The reaction mixture was incubated for 1 h at 40°C and the absorbance was determined at 525 nm. Total ascorbate was measured following the same procedure described above, except that the 100 μl of the homogenate were previously incubated with 5 μl of 100 mM dithiotreitol (DTT) for 30 min at room temperature. DTT was subsequently inactivated by addition of 5-μl 5% (w/v) N-ethylmaleimide and the absorbance was determined at 525 nm with a spectrophotometer (U-1800, HITACHI, Tokyo, Japan). The concentration of DHA was estimated from the differences in total ascorbate. The calibration curve was prepared with 0.02–0.1 μmoles of AsA and the same reaction mixture.

For the assays of GSH and oxidized glutathione (GSSG), 0.05 g samples (FW) were ground in liquid nitrogen with a pestle and mortar. The homogenates were added to 500 μl of 5% (w/v) sulfosalicylic acid and then centrifuged at 4°C for 10 min at 15,000 ×*g*. The homogenate was neutralized with 1.5 volumes of 0.5-M phosphate buffer at pH 7.5. Total glutathione (GSH + GSSG) was detected by the addition of 100 μl of neutralized homogenate to a reaction mixture containing 100-mM phosphate buffer at pH 7.5, 0.15-mM NADPH, 60-μM dithio-bis-nitrobenzoate and 0.66 U of GR (Sigma, St. Louis, USA) in a final volume of 1 ml. The reaction mixture was incubated for 1 h at 37°C and the absorbance was determined at 412 nm. GSSG was detected following the same procedure described above except that the 100 μl of the neutralized homogenate were previously incubated with 20-μl 2-vynilpyridine 1 M for 1 h at room temperature. GSH was estimated as the difference between total glutathione and GSSG. The calibration curve was prepared with 0.005–0.1 μmoles of GSH in the same reaction mixture. The data are presented as means ± SD of four independent experiments.

### Transcriptional Analysis

RNA extraction and qRT-PCR analysis were performed as described by Uji et al. (2019). Total RNA was extracted using the RNeasy Plant Mini Kit (Qiagen, Hilden, Germany) following the manufacturer's instructions. The extracted RNA was purified with the TURBO DNA-free kit (Invitrogen/Life Technologies, Carlsbad, CA) to obtain DNA-free RNA. First-strand cDNA was synthesized from 0.5 μg of total RNA using the PrimeScript II 1st strand cDNA Synthesis Kit (TaKaRa Bio, Shiga, Japan). For qRT-PCR analysis, the cDNA was diluted 10-fold and 1.0 μl of the diluted cDNA was used as a template in a 20-μl reaction volume using SYBR^®^ Premix Ex Taq™ GC (TaKaRa Bio) following the manufacturer's instructions. Real-time PCR was then performed with a LightCycler^®^ 480 System (Roche Diagnostics, Basel, Switzerland) under the following conditions: 30 s at 95°C followed by 40 cycles of 5 s at 95°C and 31 s at 60°C. The mRNA levels were calculated based on a standard curve and were normalized to levels of the 18S ribosomal RNA (*Py18SrRNA*) gene (Uji et al., 2016). The standard curve for each primer set was prepared by plotting serial cDNA dilutions (1:10 to 1:10^5^) against the threshold cycle (CT). The relative expression level was calculated as a ratio of the mRNA level to the transcription level at 0 d after ACC treatment. All the experiments were performed in triplicate. **Table S1** lists the primers that were used in this study and sequences of the analyzed genes were retrieved from *P. yezoensis* genome sequence data (Nakamura et al., 2013).

### Statistical Analysis

Data are expressed as the means ± standard deviation (SD) and analyzed using Mann-Whitney's U test for treatments with and without ACC or ACC analogs. For all analyses, *p* < 0.05 was considered statistically significant.

## Results

**Figure 2:**
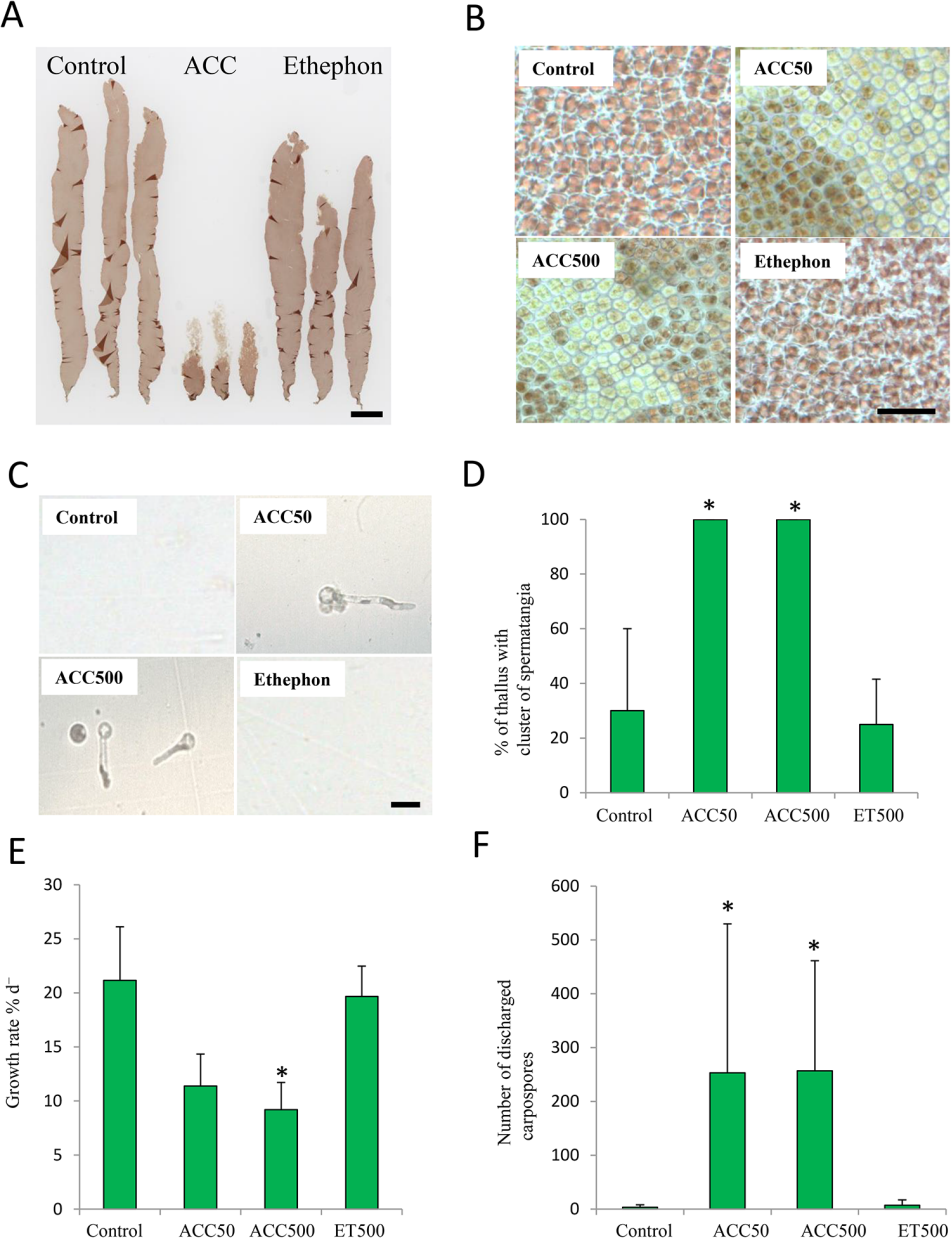
Promotion of sexual reproduction in *Pyropia yezoensis* gametophytes by1-aminocylopropane-1-carboxylic acid (ACC) but not ethephon (ET). **(A)** Gametophytes cultured in medium containing 0 or 500 μM ACC, or 500 μM ET. The thalli treated with ACC formed many spermatangia in the upper regions, which were clear or discolored. Scale bar = 10 mm. **(B)** Microscopic view of upper parts from gametophytes in medium with containing 0 or 50, 500 μM ACC, or 500 μM ET. Scale bar = 50 μm. **(C)** Microscopic view of carpospores accumulation at the bottom of culture flasks. Scale bar = 10 μm. **(D)** Formation rate of the clusters of spermatangia of gametophytes after culture with 0, 50, or 500 μM ACC, or 500 μM ET. **(E)** Growth rate of gametophytes cultured with 0, 50, or 500 μM ACC, or 500 μM ET. **(F)** The number of carpospores released from gametophytes cultured with 0, 50, 500 μM ACC, or 500 μM ET. Data are expressed as means ± SD of four independent experiments with five thalli for each condition. Asterisks indicate significant differences at *P* < 0.05 between the controls and treatments.

Firstly, we compared the effects of ethephon and ACC on growth and gametogenesis in *P. yezoensis* to examine the possible function of ACC as a signaling molecule. ACC dramatically repressed growth and promoted the formation of colorless spermatangia on the upper parts of thalli, whereas there were no significant differences between thalli treated with or without ethephon (**Figures 2A, B**). In addition, carpospores were released from the upper parts of the gametophytes treated with ACC (**Figure 2C**). All (100%) thalli of gametophytes treated with 50-μM or 500-μM ACC formed spermatangia clusters; however, only 25.0% or 30.0% of the thalli produced them when gametophytes were treated with or without (control) ethephon, respectively (**Figure 2D**). The growth rates of gametophytes cultured in media containing 50 and 500 μM ACC were 11.4% d^-1^ and 9.2% d^-1^, respectively, whereas those of gametophytes grown with ethephon or without treatment were 19.7% d^-1^ or 21.2% d^-1^, respectively (**Figure 2E**). The number of carpospores released from the gametophytes treated with ACC was ca. 250, whereas that of gametophytes treated with ethephon or without treatment was 7 or 3, respectively (**Figure 2F**).

**Figure 3:**
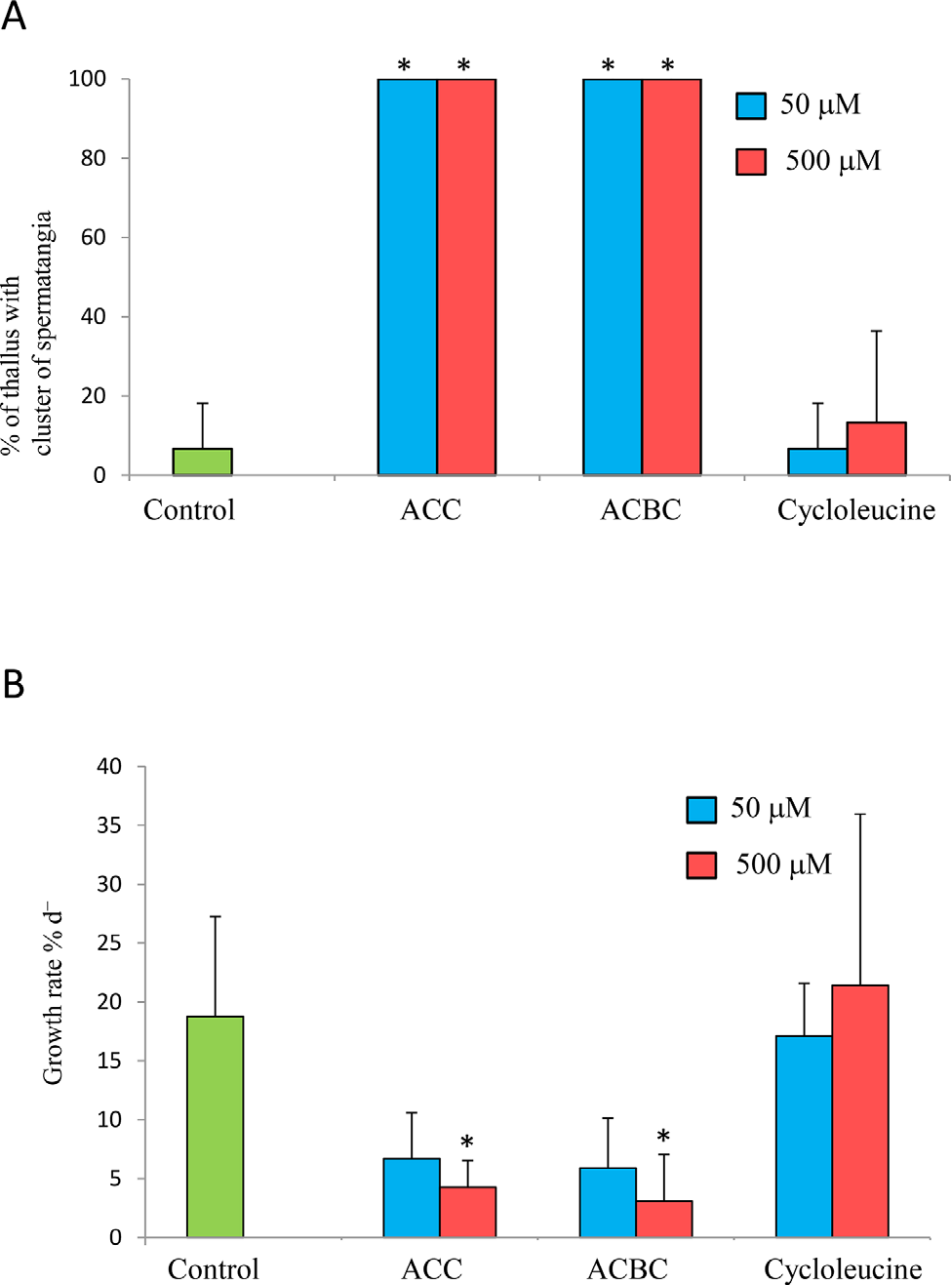
Effect of 1-aminocylopropane-1-carboxylic acid (ACC) analogs on sexual reproduction in *Pyropia yezoensis* gametophytes. **(A)** Formation spermatangia clusters on gametophytes during 10 days of culture with 1-aminocyclobutane-1-carboxylic acid (ACBC) or cycloleucine. **(B)** Growth rate of gametophytes during 10 days of culture with ACBC or cycloleucine. Data are expressed as means ± SD of four independent experiments with five thalli for each condition. Asterisks indicate significant differences at *P* < 0.05 between controls and treatments.

Next, we examined whether ACC analogs promote spermatogenesis in gametophytes of *P. yezoensis*. All *P. yezoensis* gametophytes that were cultured for 10 days in the presence of ACC or ACBC formed spermatangia (**Figure 3A**). In contrast, only 6.6% or 13.3% of thalli formed spermatangia when supplemented with 50 or 500 μM cycloleucine, respectively (**Figure 3A**). In addition, ACBC treatment inhibited the growth of gametophytes, in comparison with cycloleucine or non-ACC analog treatments, which did not (**Figure 3B**). Gametophytes cultured in media containing 50 and 500 μM ACC exhibited growth rates of 6.6% d^-1^ and 4.2% d^-1^, respectively, while those of gametophytes grown under ACBC treatment were 5.9% d^-1^ and 3.1% d^-1^, respectively. In contrast, the growth rates of gametophytes cultured in media containing 50-μM and 500-μM cycloleucine were 17.0% d^-1^ and 21.3% d^-1^, respectively.

**Figure 4:**
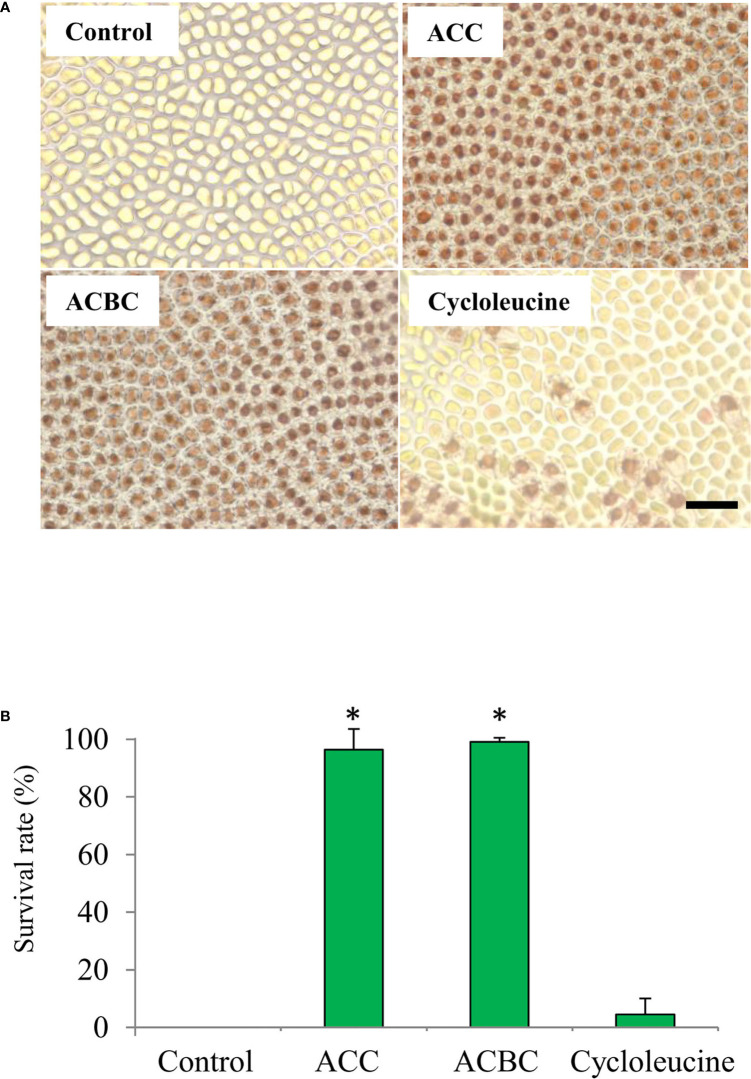
Effect of 1-aminocylopropane-1-carboxylic acid (ACC) analogs on tolerance to oxidative stress in *Pyropia yezoensis* gametophytes. **(A)** Magnified view of gametophytes subjected to 2 mM H_2_O_2_ (oxidative stress) after treatment with 0 (control) or 500 μM ACC, 500 μM 1-aminocyclobutane-1-carboxylic acid (ACBC), or 500 μM cycloleucine. Scale bar = 50 μm **(B)** The survival rate of gametophytes subjected to 2 mM H_2_O_2_ (oxidative stress) after treatment with 0 (control) or 500 μM ACC, 500 μM ACBC, or 500 μM cycloleucine. Data are expressed as means ± SD of three independent experiments with five thalli for each condition. Asterisks indicate significant differences at *P* < 0.05 between controls and treatments.

We also investigated whether ACC analogs function in oxidative stress tolerance, because gametophytes treated with ACC show enhanced tolerance to oxidative stress (Uji et al., 2016). In our experiments, thalli treated with 500 μM ACC or 500 μM ACBC demonstrated a high rate of survival at 7 days-post treatment (ACC: 96.4%, ACBC: 99.1%) in 2 mM H_2_O_2_ (**Figure 4**). However, thalli that were treated with 500 μM cycloleucine or left untreated (controls) exhibited a low rate of survival at 7-day posttreatment (control: 0.0%, cycloleucine: 4.5%) in 2 mM H_2_O_2_ (**Figure 4**). These results indicate that exogenously applied ACBC promoted sexual reproduction and enhanced the antioxidant capacity of the gametophytes at the same level as ACC but cycloleucine did not.

**Figure 5:**
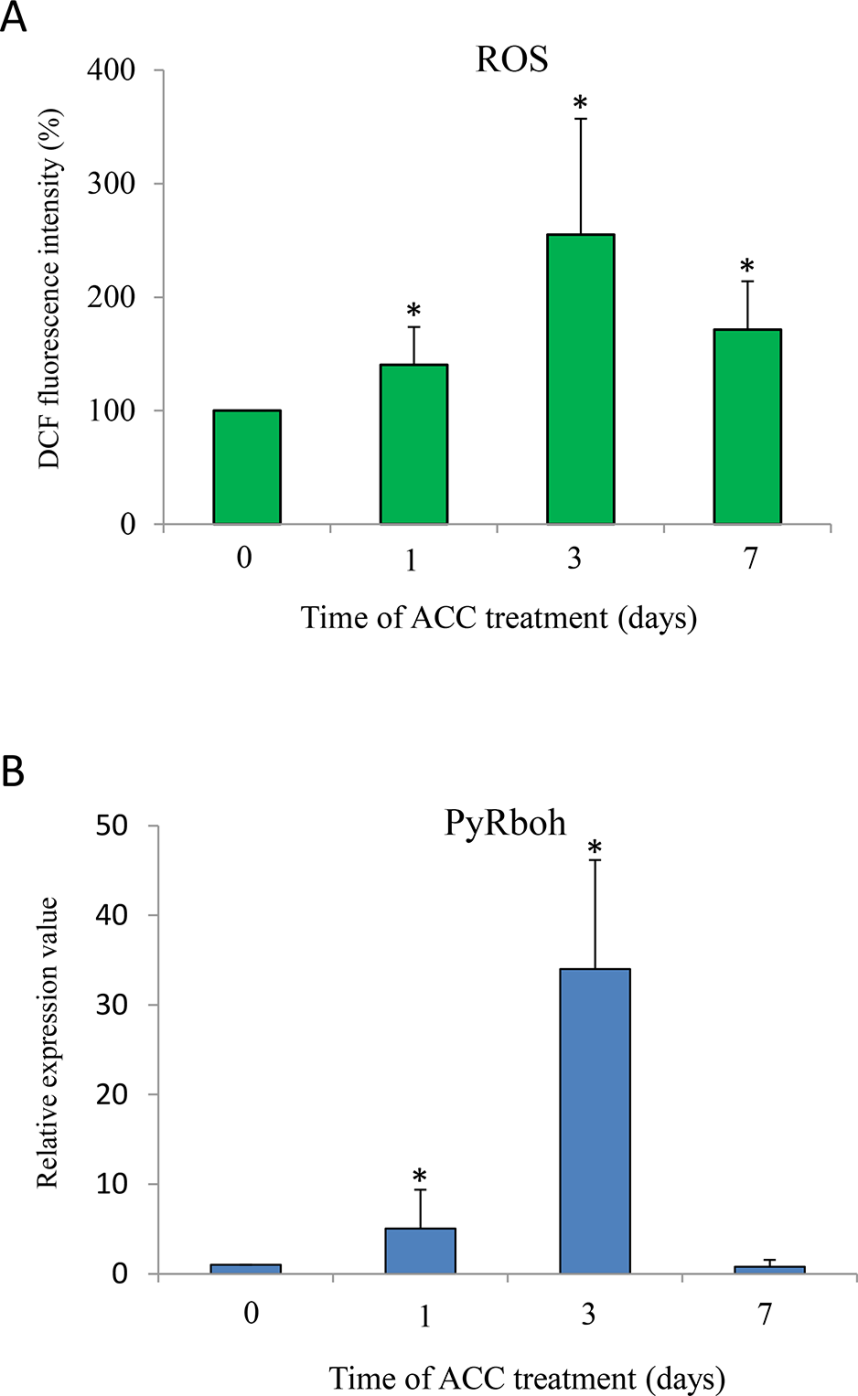
1-aminocylopropane-1-carboxylic acid (ACC) induced production of reactive oxygen species (ROS) *via* NADPH oxidase in *Pyropia yezoensis* gametophytes. **(A)** Time course of ACC-induced ROS generation in gametophytes. Gametophytes were subjected to 5 μM 2',7'-dichlorofluorescein diacetate (DCFH-DA) after treatment with ACC for 1, 3, or 7 d. The results are presented as the relative fluorescence intensity compared with that of nontreated gametophytes (0 d). Asterisks indicate significant differences at *P* < 0.05 between controls and treatments. The data are presented as means ± SD of four independent experiments. **(B)** Relative expression levels of respiratory burst oxidase homolog in *P. yezoensis* (*PyRboh*) gene in gametophytes in response to ACC. Asterisks indicate significant differences at *P* < 0.05 between controls and treatments. The data are presented as means ± SD of three independent experiments.

To determine a possible role for ROS in ACC signaling, we determined the production of intracellular ROS during ACC treatment using a ROS sensor, DCFH-DA. ACC treatment increased the DCF fluorescence within 1 d (1.40-fold) and peaked at 3 d (2.55-fold), following which it decreased at 7 d (1.71-fold) (**Figure 5A**). In addition, we tested whether Rboh activity is involved in ROS generation during ACC treatment. The mRNA transcripts of the *PyRboh* gene increased significantly after 1 d of treatment (5.06-fold), peaked at 3 d (34.0-fold), and then decreased after 7 d (0.77-fold) (**Figure 5B**).

**Figure 6:**
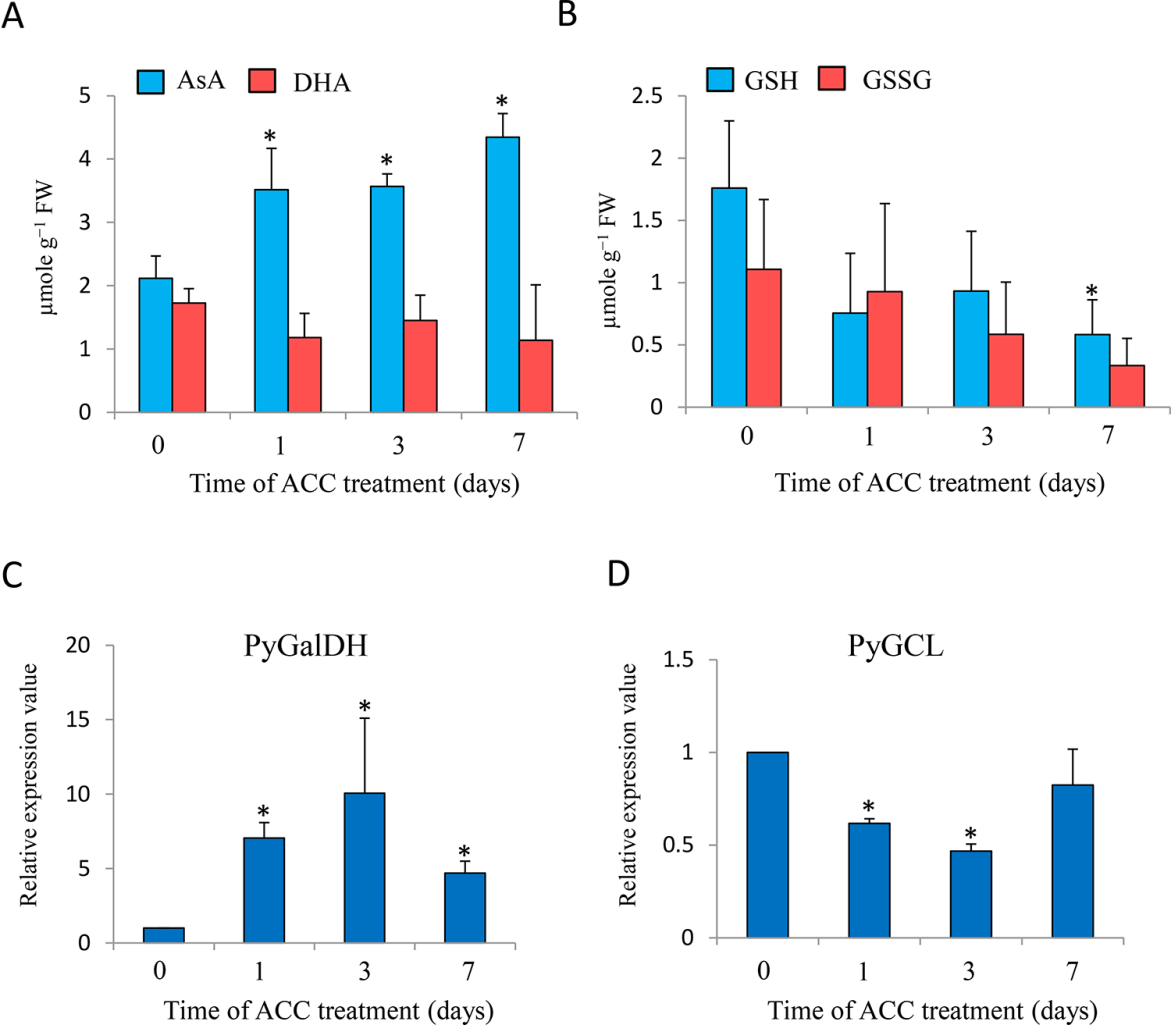
Changes in ascorbate (AsA) and glutathione (GSH) homeostasis in response to 1-aminocylopropane-1-carboxylic acid (ACC) in *Pyropia yezoensis* gametophytes. **(A)** Changes in AsA and dehydroascorbate (DHA) levels in the gametophytes in response to ACC. Compounds are expressed in micromoles per gram of fresh weight (FW). The data are presented as means ± SD of four independent experiments. **(B)** Changes in GSH and oxidized glutathione (GSSG) levels in the gametophytes in response to ACC. Compounds are expressed in micromoles per gram of FW. The data are presented as means ± SD of four independent experiments. **(C)** Relative expression levels of galactono-1,4-lactone dehydrogenase from *P. yezoensis* (PyGalDH) gene in the gametophytes in response to ACC. **(D)** Relative expression levels of glutamate cysteine ligase from *P. yezoensis* (PyGCL) gene in the gametophytes in response to ACC. The data are presented as means ± SD of three independent experiments. Asterisks indicate significant differences at *P* < 0.05 between controls and treatments.

To examine the involvement of the redox state in ACC signaling, we determined the effects of ACC application on the redox state of AsA and GSH (**Figure 6**). The AsA content of gametophytes significantly increased (1.66-fold) after 1 d of ACC treatment and peaked at 7 d (2.06-fold), but the content of DHA was not significantly changed during ACC treatment (**Figure 6A**); however, the levels of GSH and GSSG gradually decreased and the GSH content after 7 d of ACC treatment was significantly lower than that of 0 d of ACC treatment (control) (**Figure 6B**). Consistent with the content of AsA, the mRNA transcripts of *PyGalLDH*, encoding galactono-1,4-lactone dehydrogenase, which catalyzes the final step in the synthesis of AsA in higher plants, increased significantly after 1 d of ACC treatment (7.04-fold), peaked at 3 d (10.0-fold), and then decreased gradually after 7 d (4.68-fold) (**Figure 6C**). Conversely, the exogenous application of ACC resulted in the down-regulation of *PyGCL*, encoding glutamate cysteine ligase (also known as γ-glutamylcysteine synthetase), which catalyzes the rate-limiting step in the formation of GSH (**Figure 6D**). The results indicate that ACC strongly influences the cellular redox state by regulating the synthesis of AsA and GSH.

**Figure 7:**
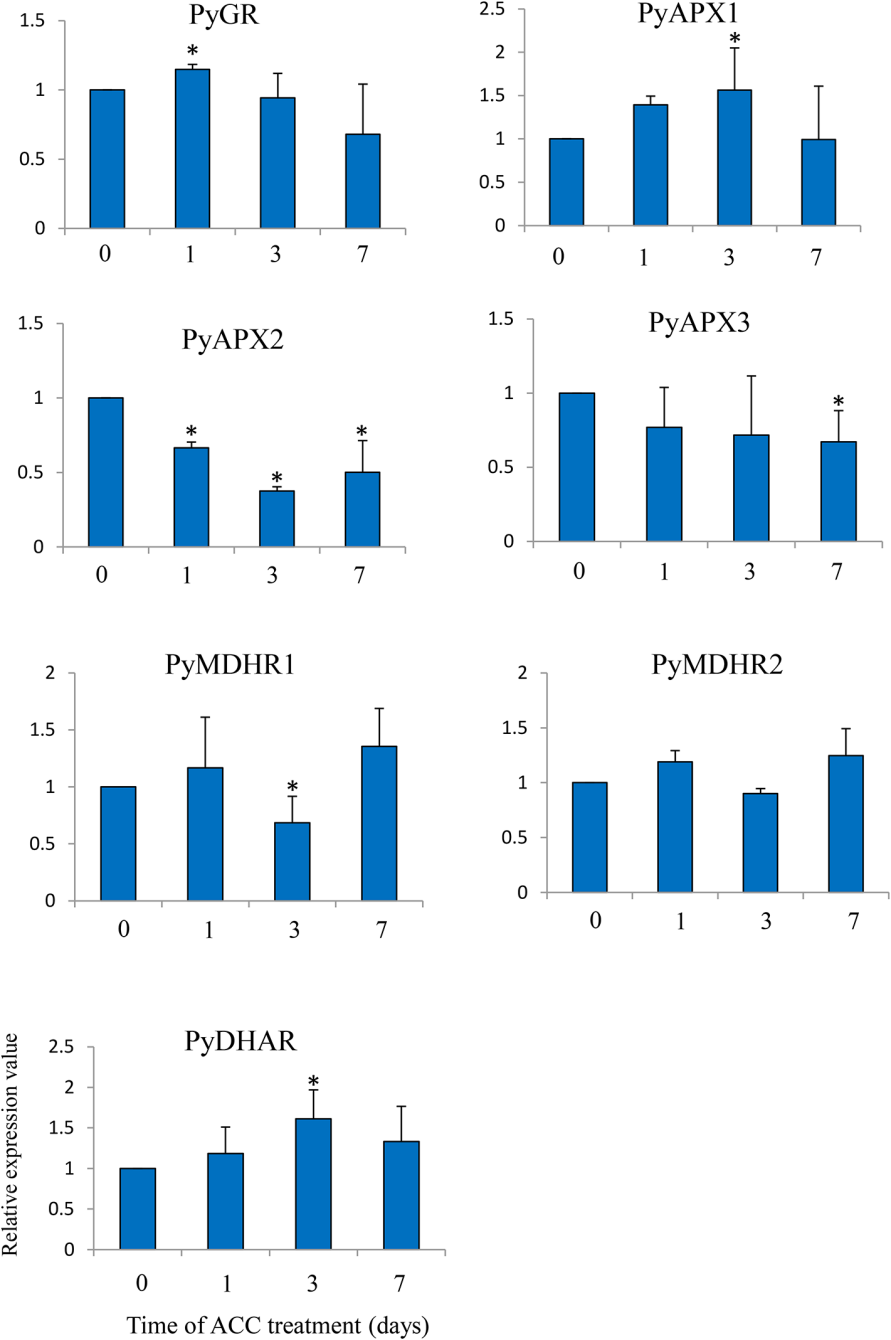
Relative expression levels of genes associated with the ascorbate-glutathione (AsA-GSH) cycle in *Pyropia yezoensis* gametophytes in response to ACC. The data are presented as means ± SD of three independent experiments. Asterisks indicate significant differences at *P* < 0.05 between controls and treatments.

Lastly, we examined the expression of genes involved in the AsA/GSH cycle under ACC treatment (**Figure 7**). There was no major fluctuation in the expression of the genes, but the expression levels of *PyGR*, *PyAPX1*, and *PyDHAR*, were slightly induced in the thalli under ACC treatment (1.14-fold –1.61-fold), while the expression levels of *PyAPX2*, *PyAPX3*, and *MDHAR1* were repressed under ACC treatment (0.37-fold–0.68-fold).

## Discussion

Ethylene is the gaseous plant hormone regulating many aspects of plant growth and development in higher plants (Lin et al., 2009; Van De Poel et al., 2015). In contrast to higher plants, information about the physiological role of ethylene in macroalgae is scarce. For example, the application of exogenous ethylene exhibited a loss of chlorophyll *a* (Chl *a*) in the green macroalga *Ulva intestinalis* (Plettner et al., 2005) and the ethylene exposure of red macroalga, *Pterocladiella capillacea*, thalli promoted the maturation of tetrasporangia (Garcia-Jimenez and Robaina, 2012). Our previous studies revealed that the ethylene precursor ACC promotes the formation of male and female gametes in *P. yezoensis* and *P. pseudolinearis* (Uji et al., 2016; Yanagisawa et al., 2019). In the present study, we found that there was no significant difference in sexual reproductive activity between gametophytes that have or have not been treated with ethephon, which converts to ethylene, is a widely used chemical replacement for ethylene treatment in higher plants (Zhang et al., 2010), whereas the ACC analog, ACBC, accelerated sexual reproduction in *P. yezoensis* in the same manner as ACC. These results suggest that ACC itself can act as a signal in red algae. ACC has also been reported to act as a primary regulator of plant growth and development independently to ethylene in higher plants (Tsuchisaka et al., 2009; Vanderstraeten and Van Der Straeten, 2017). A recent study demonstrated that ACC, but not ethylene, positively modulates guard mother cells of *A. thaliana* by controlling the expression of cell cycle regulators such as A-type cyclin and B-type cyclin-dependent kinase (Yin et al., 2019). Our previous study revealed that the application of ACC increases transcripts of homologous genes to U-type cyclin and Aurora kinase in *P. yezoensis* gametophytes (Uji et al., 2016). Thus, ACC might be a regulator of the cell cycle and cell division in plants.

Characterization of the genes disrupted in mutants is leading to the discovery of ethylene signal transduction pathway components in higher plants (Ju and Chang, 2015). In contrast, the molecular mechanism by which the ACC signaling pathway acts *via* the ACC receptor and downstream signaling components remains unclear. The majority plant hormone functions are tightly linked with ROS-mediated signaling (Xia et al., 2015; Mittler, 2017; Noctor et al., 2018) and Tsang et al. (2011) hypothesized that the production of ROS regulated by auxin might be required downstream of ACC signaling to control root elongation in higher plants. For macroalgae, knowledge about the interplay between ROS and plant hormones in development and stress responses remains scarce. However, a few studies suggest that methyl jasmonate-induced ROS play a crucial role in the defense response, conferring resistance against algal endophytes by modulating Rboh activity in red and brown algae (Collén et al., 2006; Hervé et al., 2006: Küpper et al., 2009). ROS generation in the gametophytes treated with ACC was accompanied by an increase in *PyRboh* transcripts, indicating that Rboh-mediated ROS production plays an important role in ACC-induced gametogenesis and tolerance to oxidative stress in *P. yezoensis*.

The AsA-GSH cycle is a major pathway for scavenging ROS, which consist of two antioxidants, AsA and GSH, and four antioxidant enzymes, namely, APX, MDHAR, DHAR, and GR (Pandey et al., 2015). In higher plants, the concentrations of AsA and GSH and the expression of antioxidant enzymes can be tightly regulated under abiotic and biotic stress conditions (Foyer and Noctor, 2011). The AsA content in *P. yezoensis* gametophytes greatly increased during ACC treatment, whereas the GSH content decreased gradually. However, there was no major fluctuation in the expression of genes encoding antioxidant enzymes involved in the AsA-GSH cycle. Thus, the role of the AsA-GSH cycle in redox modulation of ACC signaling remains unclear in *Pyropia*. In addition to the AsA-GSH cycle, AsA plays a protective role in photoinactivation by serving as a photosystem II (PSII) electron donor, which can alleviate photodamage, a rapid inactivation and degradation of PSII reaction centers, by accumulating ROS under heat and light stress in higher plants and green algae (Tóth et al., 2009; Tóth et al., 2011). The gametophytic thalli of *P. yezoensis* generally produce spermatia and carpogonia at the beginning of spring and, after fertilization, carpospores germinate into sporophytes, which grow in the summer when exposed to high temperatures and strong light. These findings suggest that the increase of AsA during sexual reproduction is critical to the acclimation to the habitat required for the sporophytic stage and may protect against the photoinactivation of PSII.

In contrast to AsA, the content of GSH declined in the gametophytes of *P. yezoensis* exposed to ACC in a time-dependent manner. GSH is a transducer that integrates environmental information into the cellular network, thereby affecting protein structure and activity through changes in the thiol-disulfide balance (Noctor et al., 2012). In higher plants, GSH depletion significantly increases the redox potentials of the nucleus and cytosol and influences root development in *Arabidopsis* by regulating gene expression linked to altered hormone responses (Schnaubelt et al., 2015). In addition, abscisic acid and methyl jasmonate decrease GSH contents, which lead to enhanced stomatal closure in *A. thaliana* (Okuma et al., 2011; Akter et al., 2012). There has been no report of roles for GSH in development or plant hormone signaling in red algae, but the results of this study suggest that alterations in the glutathione status participate in the signal transduction cascades of the ACC response during sexual reproduction in *Pyropia*.

The L-galactose pathway is considered to be the principal pathway for synthesis of AsA in higher plants. The final step, the oxidation of L-galactono-1,4-lactone into AsA, is catalyzed by GalLDH (Smirnoff, 2000). A number of reports suggest that GalLDH is an important regulatory enzyme in the accumulation of AsA in higher plants (Tamaoki et al., 2003; Pateraki et al., 2004; Rodriguez-Ruiza et al., 2017). Wheeler et al. (2015) suggested that multicellular red algae use the AsA biosynthetic pathway of higher plants, except that they employ an unidentified enzyme to generate L-galactose from GDP-L-galactose instead of GDP-L-galactose phosphorylase (VTC2). In the present study, we found that the exogenous application of ACC significantly increased the content of AsA, which accompanied higher levels of *PyGalLDH* expression (**Figure 6C**). The results suggest that GalLDH is the rate-limiting enzyme for AsA synthesis in red algae.

ACC, ACBC, and cycloleucine bind to NR1, the glycine binding subunit of the ionotropic glutamate receptor (iGluR), which is the N-methyl-D-aspartate (NMDA) receptor in mammals and is important to fast excitatory synaptic transmission (Nahum-Levy et al., 1999; Sheinin et al., 2002; Inanobe et al., 2005); however, ACC and ACBC are partial agonists and cycloleucine is an antagonist and they have dramatically different affinities for the NR1 ligand binding core. Displacement experiments indicate that ACC binds with a 5.5-fold higher affinity than glycine and ACBC and cycloleucine bind much more weakly than glycine with 31-fold and 580-fold lower affinities, respectively (Inanobe et al., 2005). In the present study, ACBC induced gametogenesis and enhanced tolerance to oxidative stress at the same level as ACC, suggesting that the affinity of ACBC on an unidentified ACC receptor may be equal to ACC affinity. In contrast, cycloleucine has no effect on the promotion or inhibition of sexual reproduction, suggesting that cycloleucine is neither an agonist nor antagonist to the ACC receptor in *P. yezoensis*.

The activation of the NMDA receptor from mammals is required for the binding of both glycine and glutamate (Johnson and Ascher, 1987). As described above, ACC functions as a partial agonist at the glycine site of the NMDA receptor. Furthermore, the role of ACC and GLRs in root morphogenesis, as well as the strong expression of GLRs in root hair systems in *A. thaliana*, suggest that ACC is likely to be a ligand of plant GLRs on the ligand binding domain in higher plants (Le Deunff and Lecourt, 2016). In terms of red algal GLRs, two GLR homologs have been found in the genomes of the multicellular red algae *Porphyra umbilicalis* and *Gracilariopsis chorda*. In addition, we found two GLR homologs that showed significant similarity to *Branchiostoma* and *Octopus* in the draft *P. yezoensis* genome sequence (Data not shown). There is no information on the characterization of GLRs from red algae or amino acid signaling, but PyGLRs may be involved in the ACC-based signaling regulation of sexual reproduction in *P. yezoensis*. Intriguingly, our recent study demonstrated that exogenous glycine or glutamate promoted sexual reproduction by inducing ACC-responsive genes (Onodera et al. unpublished). Thus, further research on the relationship between ACC and PyGLRs is required to elucidate the mechanisms that regulate the switch between the vegetative and sexually reproductive phases in *P. yezoensis*.

In conclusion, we show that ACC stimulates the formation of sexual cells and protects against oxidative stress in gametophytes of *P. yezoensis* independently of ethylene signaling. ACC regulates the redox state generating ROS through NADPH oxidase and antioxidants such as AsA and GSH during sexual reproduction. These findings provide new insights into not only the regulation of the red algae life cycle but also the evolutionary perspective of the functions and signaling of plant hormones.

## Data Availability Statement

All datasets generated for this study are included in the article/**Supplementary Material**.

## Author Contributions

TU was responsible for the design of the experiments, and interpretation of data. TU and HE performed the experiments. TU and HM wrote the manuscript. All authors have read and approved the final manuscript.

## Funding

This study was supported by a grant-in-aid for Young Scientists (B) (16K18740 to TU) and Young Scientists (19K1590709 to TU) from the Japan Society for the Promotion of Science.

## Conflict of Interest

The authors declare that the research was conducted in the absence of any commercial or financial relationships that could be construed as a potential conflict of interest.
