# Corrigendum: Sexual Reproduction *via* a 1-Aminocyclopropane-1-Carboxylic Acid-Dependent Pathway Through Redox Modulation in the Marine Red Alga *Pyropia yezoensis* (Rhodophyta)

**DOI:** 10.3389/fpls.2020.609686

**Published:** 2020-10-30

**Authors:** Toshiki Uji, Harune Endo, Hiroyuki Mizuta

**Affiliations:** Laboratory of Aquaculture Genetics and Genomics, Division of Marine Life Science, Faculty of Fisheries Sciences, Hokkaido University, Hakodate, Japan

**Keywords:** *Pyropia*, red algae, sexual reproduction, 1-aminocylopropane-1-carboxylic acid, redox signaling, ethylene, plant hormone

In the original article, there was a mistake in Figure 4A as published. The panel (ACC) has been changed. The corrected Figure 4A appears below.

**Figure 4:**
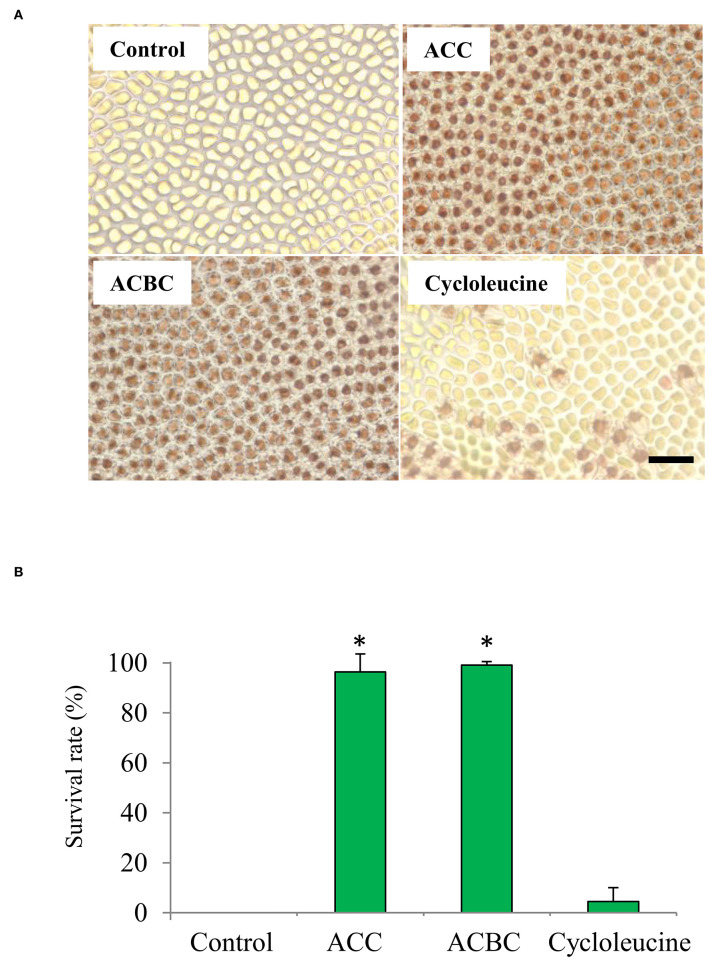
Effect of 1-aminocylopropane-1-carboxylic acid (ACC) analogs on tolerance to oxidative stress in *Pyropia yezoensis* gametophytes. **(A)** Magnified view of gametophytes subjected to 2 mM H_2_O_2_ (oxidative stress) after treatment with 0 (control) or 500 μM ACC, 500 μM 1-aminocyclobutane-1-carboxylic acid (ACBC), or 500 μM cycloleucine. Scale bar = 50 μm. **(B)** The survival rate of gametophytes subjected to 2 mM H_2_O_2_ (oxidative stress) after treatment with 0 (control) or 500 μM ACC, 500 μM ACBC, or 500 μM cycloleucine. Data are expressed as means ± SD of three independent experiments with five thalli for each condition. Asterisks indicate significant differences at *P* <0.05 between controls and treatments.

The authors apologize for this error and state that this does not change the scientific conclusions of the article in any way. The original article has been updated.

