# Supplementary material for: Sexual Reproduction via a 1-Aminocyclopropane-1-Carboxylic Acid-Dependent Pathway Through Redox Modulation in the Marine Red Alga Pyropia yezoensis (Rhodophyta)
Source: Front Plant Sci. 2020 Feb 12;11:60. doi: 10.3389/fpls.2020.00060 (PMC7028691; doi:10.3389/fpls.2020.00060)
Supplement: Supplementary file 1 [file DataSheet_1.pdf]

Table S1. The list of primers used for gene expression analysis by quantitative Real Time PCR

Primer name	Contig ID	Sequence (5’-3’)
PyRboh-F1	contig_36762_g8710	TTGTGCTCACCTTCCTGATG
PyRboh-R1	contig_36762_g8710	GCGTTCCAGTTGATGAGGTT
PyGalDH-F1	contig_27288_g6714	TTTTTGACGTGAGCCCCTAC
PyGalDH-R1	contig_27288_g6714	AGTCCCCAAACTCAATGTCG
PyGCL-F1	contig_6454_g1464	GACCTGGTGGAGCAAAACAT
PyGCL-R1	contig_6454_g1464	GAATGTTGCGCGACAGAGTA
PyGR-F1	contig_21457_g5287	CGTGTTCAAGAGCGACTTCA
PyGR-R1	contig_21457_g5287	CTAGTCGGGTGTACGCCAAT
PyAPX1-F1	contig_10207_g2440	ATCAGCTACGCGGACCTCTA
PyAPX1-R1	contig_10207_g2440	AAGCCCATCCGGTAAAAGAT
PyAPX2-F1	contig_9033_g2146	AGGTGCCCATTGTCTTTGAC
PyAPX2-R1	contig_9033_g2146	CCTTTGAATAGTCGGCGAAA
PyAPX3-F1	contig_35733_g8551	TTGGGAATGGGTACCTCAAG
PyAPX3-R1	contig_35733_g8551	GCCCAGCAACTTGACACTCT
PyMDHR1-F1	contig_11687_g2778	GCTGCCAGGTCAAGATGATT
PyMDHR1-R1	contig_11687_g2778	TCGTCACCCAGAATCTCCTC
PyMDHR2-F1	contig_2056_g353	GGTACATTGGGATGGAGGTG
PyMDHR2-R1	contig_2056_g353	ACCTCATCCTTGACGTGGTC
PyDHAR-F1	contig_4779_g1033	GTGTTCTCTCGCACCCTCTC
PyDHAR-R1	contig_4779_g1033	TGGTAAAACTCGGCCATCTC
Py18S-F1	* D79976	AGGGTTGATCCGCAGGGAAG
Py18S-R1	* D79976	GCTTGCGCCCACTCCATTAG

*Accession number in GenBank
